# The Extraction of Teeth for Parturient Women

**Published:** 1886-01

**Authors:** Garrett Newkirk

**Affiliations:** Chicago, Ill.


### THE EXTRACTION OF TEETH FOR PARTURIENT WOMEN.

BY GARRETT NEWKIRK, M. D., CHICAGO, ILL.

Some months since I saw an article relating to some one—a lady in Mississippi, I think—who believed that the extraction of a tooth had been necessary for the birth of each child. As it appeared, this idea had the endorsement of her medical adviser and others. The item has been republished in several journals, without any one, so far as I know, questioning either facts or conclusions.

Judging by past experience, in her third labor the lady believed that before it could be concluded she must lose a tooth. The idea took firm possession of her mind. The physician at first declining the extraction, the pains ceased, or became weak and inefficient for six or eight hours. Then, yielding to her solicitations, he extracted the tooth, which we are led to infer was a good one, and the child was born within an hour. Therefore, for some mysterious reason, the extraction of the tooth was the one and only thing required to terminate the labor. Verily, logic is logic.

Let us see. In the first place, when her oldest was born, she may have had an aching tooth, a tooth really diseased and causing disturbance. It is a fact well known to all physicians, that any irritating cause may disturb the rhythm and force of labor pains. Anything which distracts the mind or excites particular attention will do the same thing. The sudden appearance of an attendant, other than the one expected, may block the wheels of labor for hours. So may undigested food in the stomach, a full bladder, a rheumatic joint, a colic, a whim, a grief, a disappointment, or the mere presence of some female whom the patient dislikes. There is no end to the list of disturbing elements that may interfere with the always desired smooth and regular course of labor.

Again, there is nothing more common than the occurrence of "wandering pains." These may be in the limbs, not infrequently with cramps; also in the stomach, in the nerves and muscles of the trunk, as well as in the head and face.

After the first and second labors, nearly every woman has some positive notions of her own about herself, all rooted and grounded. That which was done in a former labor, when things turned out well, must be done again. The parturient woman is wrought up to a high pitch of mental and nervous tension. She is usually very positive, and exacting to the last detail. No queen could be more imperious, and she brooks no opposition. She is no respecter of persons, nor of opinions that conflict with her own. She is in a hurry to get through, and has no time for nice arguments and distinctions. Whatever real or fancied good she has experienced from an act in former times, she requires again. Right or wrong, she demands it, and refusal chafes and frets her. There may be nothing in the thing she wants, but give it to her and she is satisfied.

In the case cited, there was a woman who knew a tooth had to come. Opposition chafed her, and perhaps from mental disturbance alone, pains were weak for hours. Quite likely, too, the delay was more apparent than real, nature taking time to relax the circular fibres of the *os uteri,* preparatory to the true expulsive pains of the last hour.

It is not impossible that the shock of the operation may have acted as a nervous stimulant, and excited more vigorous contractions of the womb; so would a shock produced by such other means as sudden applications of heat and cold, or a few smart blows on the soles of the feet and palms of the hands.

But there is no evidence whatever, in the facts stated, to support the theory held, that the tooth, as a tooth, had anything to do with retarding that labor. It was no culprit, but an unfortunate victim, guilty of nothing worthy of death.

